# Threshold Successive Cancellation Flip Decoding Algorithm for Polar Codes: Design and Performance

**DOI:** 10.3390/e27060626

**Published:** 2025-06-12

**Authors:** Zhicheng Liu, Liuquan Yao, Shuai Yuan, Guiying Yan, Zhiming Ma, Yuting Liu

**Affiliations:** 1 School of Mathematics and Statistics, Beijing Jiaotong University, Beijing 100044, China; 20118002@bjtu.edu.cn; 2 University of Chinese Academy of Sciences, Academy of Mathematics and Systems Science, CAS, Beijing 100190, China; yaoliuquan20@mails.ucas.ac.cn (L.Y.); yuanshuai2020@amss.ac.cn (S.Y.); yangy@amss.ac.cn (G.Y.); mazm@amt.ac.cn (Z.M.)

**Keywords:** polar codes, successive cancellation decoding, threshold successive cancellation flip algorithm, delay probability

## Abstract

In this paper, we propose the threshold successive cancellation flip (Th-SCF) decoding algorithm for polar codes, which enhances the performance of the SC decoder while maintaining low complexity. Theoretical analysis reveals that Th-SCF asymptotically delays the first error position (FEP, the first part where the SC decoder fails) with probability 1, ensuring high decoding performance. Simulation results show that the Th-SCF algorithm achieves performance comparable to the dynamic SC flip (D-SCF) algorithm, but with a reduction in complexity by eliminating the need for sorting operations. A key contribution of this work is the rigorous theoretical framework supporting the Th-SCF algorithm, distinguishing it from existing SC flip (SCF) decoding methods. This theoretical foundation not only explains the performance improvements but also provides insights into the underlying mechanisms of flipping. The proposed Th-SCF algorithm demonstrates strong performance across a wide range of code lengths and rates, and its performance remains stable within a certain threshold range, indicating its practical applicability in real-world communication systems. These results offer valuable perspectives for the design of efficient flip decoding strategies in 5G and future networks.

## 1. Introduction

Polar codes, introduced by Arıkan in [1], are groundbreaking capacity-achieving codes that have fundamentally reshaped the field of coding theory. Their achievement of channel capacity through explicit construction has led to widespread practical applications, with one prominent example being their adoption in 5G control channels [2], which has spurred advancements in decoding techniques.

Among the various decoding methods for polar codes, successive cancellation (SC) decoding, initially proposed by Arıkan [1], is known for its simplicity and effectiveness. However, the performance of the SC decoder is limited for finite code lengths. To improve this, several enhancements have been introduced, such as log-likelihood ratio-based successive cancellation list (LLR-based SCL) decoding and cyclic redundancy check-aided SCL (CA-SCL) decoding [3,4,5], which improve performance by integrating additional information during the decoding process.

An alternative to standard SCL decoders is the SC flip (SCF) algorithm, initially introduced in Figure 5 of [6] and later enhanced in [7]. In [7], the authors significantly reduced the search space of unreliable bits by introducing the concept of the critical set, which consists of the first information bits of rate-1 subblocks ($R_{1}$ node) derived from the decomposed complete code tree in a polar code. The authors demonstrated that the incorrectly decoded bits are highly likely to be found within the critical set, providing a solid foundation for the design of the algorithm. While the critical set SCF algorithm (Algorithm 2 of ref. [7]) also allows multiple bits to be flipped per iteration, it employs a progressive, multi-level bit-flipping strategy, setting it apart from the D-SCF algorithm [8], which flips multiple potentially erroneous bits simultaneously in one iteration.

The SCF decoder improves upon the basic SC decoder but still falls short compared to SCL decoders with moderate list sizes [8]. To bridge this gap, the D-SCF algorithm [9] extends SCF by combining bit reliability with its position in the information set. While the multi-flip strategy in the D-SCF algorithm enhances performance by flipping multiple bits per iteration, its increased complexity limits its practical applicability [9].

The algorithm proposed in [10] also employs a threshold-based approach similar to ours, but the key innovation of our proposed method lies in its solid theoretical foundation. While previous works primarily relied on experimental observations—such as the significant difference in LLR magnitudes between erroneous and correct bits—our approach provides a rigorous theoretical explanation for the effectiveness of the threshold-based SCF algorithm in correcting errors in SC decoding. This theoretical framework offers a comprehensive evaluation of the performance of the Th-SCF algorithm, distinguishing it from earlier strategies.

Given that point-to-point communication forms the theoretical foundation of network information theory [11,12,13,14,15], enhancing classical decoding techniques (such as successive cancellation (SC) decoding for polar codes) with solid theoretical guarantees is essential. Although SCF decoding algorithms have shown promising empirical results, their development has been hindered by the absence of a rigorous theoretical foundation, as most approaches rely heavily on simulations. Several key questions remain unanswered, and resolving them will provide crucial insights into the underlying mechanisms of the flip algorithm. Specifically, how does the flipping technique correct errors in SC decoding, and how effective is it in doing so? This work seeks to address these questions, which are essential for the design of practical and efficient flip decoding algorithms.

In this work, we propose a threshold SCF (Th-SCF) decoding algorithm with provable theoretical properties. The key contributions of this work are as follows:

- We prove that, asymptotically, the Th-SCF algorithm delays the first error position (FEP) with probability 1 (Theorem 1). This result demonstrates that the Th-SCF algorithm effectively improves the SC decoding performance by delaying the FEP, leading to a substantial enhancement in error correction efficiency.
- We propose a novel flip algorithm called the Th-SCF algorithm (Algorithm 1), based on our theoretical analysis (Theorem 1), which achieves performance comparable to the D-SCF algorithm using a single bit-flip for CRC-aided polar codes. This approach not only enhances error-correction performance with provable theoretical guarantees but also ensures practical implementation feasibility.

The remainder of the paper is structured as follows: Section 2 provides an overview of polar codes and the SC/SCL decoding algorithms. In Section 3, we derive the delay probability for the first error position (FEP) in the Th-SCF algorithm. Section 4 presents simulation results that demonstrate the performance of the proposed algorithm. Finally, Section 5 concludes the paper.

*Notation Conventions:* This paper defines the probability density function (PDF) of *X* as $P_{X}\left(x\right)$, with P(·) the probability measure, Z(W) and I(W) representing the Bhattacharyya parameter and symmetric capacity of channel *W*, respectively, and sgn(·) denoting the sign function. The Gaussian-*Q* function is defined as $Q\left(x\right)=\frac{1}{\sqrt{2\pi }}\int_{x}^{\infty }e^{-t^{2}/2}dt$, its inverse is denoted by $Q^{-1}\left(y\right)≜\left\{x:Q\left(x\right)=y\right\}$, and the cumulative distribution function (CDF) of the standard normal distribution is expressed as Φ(x)=1−Q(x). Logarithms are expressed in base 2 as $\log_{2}\left(\cdot \right)$, unless otherwise specified. The natural logarithm is denoted by ln(·). Matrices and vectors are denoted in boldface.

## 2. Brief Review of the Encoding and Decoding Algorithms of Polar Code

In this section, we provide a comprehensive overview of the theoretical foundations necessary for understanding and analyzing polar codes and their decoding algorithms. In Section 2.1, we introduce the basic principles and mathematical formulation of polar codes. Section 2.2 reviews the successive cancellation (SC) and successive cancellation list (SCL) decoding algorithms, with a focus on the computation of log-likelihood ratio (LLR) sequences. Finally, we examine key concepts related to SCF decoding, including the min-LLR SCF, D-SCF and simplified D-SCF algorithms, along with their respective implementation strategies in Section 2.3. Together, these discussions establish a rigorous theoretical basis for the performance analysis and algorithmic development presented in subsequent sections.

### 2.1. Generating Polar Codes—An Overview

Polar codes of length $N=2^{n}$ are constructed through the following encoding process:

(1) $$x_{0:N}=u_{0:N}G_{N},$$

where $x_{0:N}=\left[x_{0},\cdots ,x_{N-1}\right]$ represents the encoded bits, $u_{0:N}=\left[u_{0},\cdots ,u_{N-1}\right]$ denotes the source bits, and $G_{N}=B_{n}F^{⊗n}$ is the generator matrix. Here, $F^{⊗n}$ represents the *n*-th Kronecker power of the matrix $F=\left[\begin{array}{c} \begin{array}{cc} 1 & 0\\ 1 & 1 \end{array} \end{array}\right],$ which is the fundamental building block of polar codes, and $B_{n}$ is the bit-reversal permutation matrix as described in Equation (70) of ref. [1].

A CRC-aided polar code is an extended version of the polar code [6], constructed using a similar approach to that of standard polar codes.

**Figure 1:**
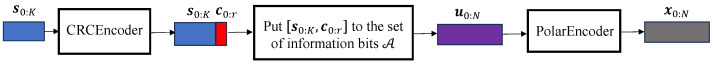
Construction flowchart of CRC-aided polar codes.

In Figure 1, $s_{0:K}=\left\{s_{0},\cdots ,s_{K-1}\right\}$ (the blue-colored sequence) represent the *K* source bits, while $c_{0:r}=\left\{c_{0},\cdots ,c_{r-1}\right\}$ (the red-colored sequence) are the *r* cyclic redundancy check (CRC) bits. These bits are then combined as $\left[s_{0:K},c_{0:r}\right]=\left[s_{0},\cdots ,s_{K-1},c_{0},\cdots ,c_{r-1}\right]$ and placed into the information bit set A. The resulting sequence $u_{0:N}$ (the brown-colored sequence) is then encoded using the method shown in Equation (1) to generate the encoded codeword $x_{0:N}$ (the sequence in purple). The notation (N,K+r) is used in this work to indicate a polar code of length *N*, *K* information bits, and *r* additional CRC bits.

Channel polarization is a process by which the channel is transformed into a set of channels that are either noiseless or fully noisy. To achieve reliable communication, the *K* most reliable bit positions (out of the *N* total positions, indexed from 0 to N−1) are selected to carry information bits. The set of information bits is denoted by A, while the remaining N−K indices are reserved for frozen bits, which are set to fixed values (typically zero). The rate of the code is given by $R=\frac{K}{N}$, where *K* is the number of information bits and *N* is the total code length.

The encoded bits ${\left\{x_{i}\right\}}_{i=0}^{N-1}$ are then mapped to binary phase shift keying (BPSK) symbols and transmitted over an additive white Gaussian noise (AWGN) channel, i.e., a binary-input AWGN (BI-AWGN) channel denoted by *W*, then the received symbols are given by:

$$y_{i}=1-2x_{i}+n_{i},$$

where $n_{i}∼N\left(0,\sigma^{2}\right)$ represents the Gaussian noise with variance $\sigma^{2}$. The received signal vector is $y_{0:N}=\left[y_{0},\ldots ,y_{N-1}\right]$, which is used for decoding at the receiver.

This framework for polar code construction ensures that the code’s performance approaches the channel capacity as the code length *N* grows, especially when combined with efficient decoding algorithms such as SC algorithm and its list-based variants.

### 2.2. SC/SCL-Based Decoding for Polar Codes

In this section, we provide an overview of the SC decoding process, which is a key decoding method for polar codes. We begin by explaining how the LLRs are computed. Next, we describe the recursive *f*- and *g*-functions used in SC decoding to efficiently recover the codeword. We then introduce SCL decoding, an enhancement to SC decoding that maintains multiple candidate paths, improving error correction performance, especially for finite block lengths. Finally, we present the workflow of the CRC method, which increases the probability of finding the correct decoding result, thus boosting the finite length performance of SC decoding.

1. **LLR Calculation and the f-function**
  Denote the LLR for a received $y_{i}$ as $L\left(y_{i}\right)=\frac{2y_{i}}{\sigma^{2}}$ with $\sigma^{2}$ the noise variance. For a length-2 polar code, the information bits $u_{0:2}=\left[u_{0},u_{1}\right]$ are decoded as follows:
  To compute the LLR of $u_{0}$, we use the min-sum approximation of the *f*-function, as given in Equation (10) of ref. [16]:
  $$L\left(u_{0}\right)=\mathrm{sgn}\left(L\left(y_{0}\right)\right)\cdot \mathrm{sgn}\left(L\left(y_{1}\right)\right)\cdot \min\{|L\left(y_{0}\right)|,|L\left(y_{1}\right)\left|\right\}.$$
  Then a hard decision on $L(u_{0})$ is made to obtain the estimate ${\hat{u}}_{0}$.
2. **The g-function and Update of $L(u_{1})$**
  Next, the *g*-function updates the LLR for the second information bit $u_{1}$:
  $$L\left(u_{1}\right)=\left(1-{\hat{u}}_{0}\right)L\left(y_{0}\right)+L\left(y_{1}\right),$$
  where ${\hat{u}}_{0}$ is the decision made for $u_{0}$ in the previous step. After updating $L(u_{1})$, a hard decision is made to obtain the estimate ${\hat{u}}_{1}$.
3. **Recursive f- and g-Functions in SC Decoding**
  The successive cancellation (SC) decoder recursively applies the *f*- and *g*-functions to estimate the information bits of a polar code. For a code of length $N=2^{n}$, the decoder produces an output vector ${\hat{u}}_{0:N}=\left[{\hat{u}}_{0},\ldots ,{\hat{u}}_{N-1}\right]$. Each bit ${\hat{u}}_{i}$ is decoded sequentially based on the previously decoded bits ${\hat{u}}_{0:i}=\left[{\hat{u}}_{0},\ldots ,{\hat{u}}_{i-1}\right]$ according to the following decision rule for all i∈{0,…,N−1}:
  $${\hat{u}}_{i}=\left\{\begin{array}{cc} 0, & \mathrm{if}\;L(y_{0:N},{\hat{u}}_{0:i}|u_{i})\geq 0\;\mathrm{and}\;i\in A,\\ 1, & \mathrm{if}\;L(y_{0:N},{\hat{u}}_{0:i}|u_{i})<0\;\mathrm{and}\;i\in A,\\ u_{i}, & \mathrm{if}\;i\in A^{c}, \end{array}\right.$$
  where A denotes the set of information bits, and $A^{c}$ represents the set of frozen bits. The function $L(y_{0:N},{\hat{u}}_{0:i}|u_{i})$, or abbreviated as $L_{N}^{\left(i\right)}$, is calculated as:
  $$L_{N}^{\left(i\right)}=\log\frac{W_{N}^{\left(i\right)}\left(y_{0:N},{\hat{u}}_{0:i}|u_{i}=0\right)}{W_{N}^{\left(i\right)}\left(y_{0:N},{\hat{u}}_{0:i}|u_{i}=1\right)},$$
  where $W_{N}^{\left(i\right)}$ represents the *i*-th bit channel, and $L_{N}^{\left(i\right)}$ is referred to as the LLR for the information bit $u_{i}$, as defined in Equation (5) of ref. [1].
4. **Successive Cancellation List (SCL) Decoding**
  Building upon the SC decoding framework, successive cancellation list (SCL) decoding enhances performance by maintaining a list of candidate decoding paths. Instead of making a hard decision at each information bit, the decoder explores both possible bit values and retains the *L* most reliable decoding paths based on their path metrics. This list-based approach allows the decoder to track multiple hypotheses in parallel and defer the final decision until all bits have been processed.
  At each information bit position, the decoder recursively updates the list by expanding each path into two candidates and selecting the *L* best ones according to a reliability criterion. Each path in the list represents a potential decoded sequence, and the final output is typically chosen as the most likely path, often aided by techniques such as cyclic redundancy check (CRC) for path selection [3,4,5].
  This enhancement significantly improves decoding performance over SC decoding, particularly for moderate to long block lengths. By expanding the solution space through a more thorough exploration of candidate paths based on the path metric (PM), it effectively reduces the probability of early decoding errors as demonstrated in Theorem 1 of ref. [4].
5. **Adjustments after Integrating CRC (Section IV-A of ref. [6])**
  To incorporate CRC into the decoding process, we assume we are given a polar code with rate $\tilde{R}=\frac{K}{N}$ and an information bit set $\tilde{A}$. A CRC of length *r* is added to the code to tell us, with high probability, whether an estimate ${\hat{u}}_{0:N}=\left[{\hat{u}}_{0},{\hat{u}}_{1},\ldots ,{\hat{u}}_{N-1}\right]$ obtained from the SC decoder is a valid codeword or not.
  To account for the added CRC, the rate of the polar code is effectively increased to $R=\tilde{R}+\frac{r}{N}=\frac{K+r}{N}$. This ensures that the overall information rate remains unchanged. In practice, the set of information bits $\tilde{A}$ is extended by adding the *r* most reliable channel indices from the complementary set ${\tilde{A}}^{c}$, which is denoted as ${\tilde{A}}_{r-\mathrm{most}}^{c}$. Thus, the new set of information bits becomes $A=\tilde{A}\cup {\tilde{A}}_{r-\mathrm{most}}^{c}$.
  This approach ensures that the CRC is incorporated efficiently into the decoding process, improving error detection and providing an effective way to identify valid codeword estimates.

### 2.3. Min-LLR SCF and D-SCF Decoding of Polar Codes

The min-LLR SCF algorithm Figure 5 of ref. [6] improves the performance of SC decoding by flipping the bit corresponding to the smallest absolute value of the LLR, $|L_{N}^{\left(i\right)}|$. This bit-flipping strategy targets the least reliable bits, which can help reduce errors and improve the overall decoding performance.

In comparison, the D-SCF algorithm (see Algorithm 2 of [9]), utilizes a more sophisticated metric, $M_{\alpha }\left(i\right)$, which combines the reliability of the bit with its position in the information set. This metric is designed to balance the decoding performance with the computational complexity, improving the decision process by considering both the magnitude of the LLR and the ordering of bits. The metric $M_{\alpha }\left(i\right)$ is computed as follows:

(2) $$M_{\alpha }\left(i\right)=\left|L_{N}^{\left(i\right)}\right|+\frac{1}{\alpha }\sum_{j<i,j\in A}\log\left(1+\exp\left(-\alpha |L_{N}^{\left(j\right)}|\right)\right),$$

where α is a parameter that controls the influence of the prior bits in the summation, and the sum runs over all previous information bits j∈A (those that carry information). The parameter α is optimized using Eqaution (23) of [9].

Some researchers have noted that the metric in the D-SCF algorithm can be simplified without significantly compromising performance [17]. Through numerical experiments, they found that replacing the original metric, i.e., Equation (2), with a simplified version yields performance comparable to that of the original D-SCF decoding algorithm. The simplified metric is expressed as follows:

(3) $$M_{\alpha }\left(i\right)=\left|L_{N}^{\left(i\right)}\right|+\sum_{j\in A,j<i}J\left(L_{N}^{\left(j\right)}\right),$$

where J(·) can be approximated according to [17]:

$$J\left(L_{N}^{\left(j\right)}\right)=\left\{\begin{array}{cc} 1.5, & \mathrm{if}\;\left|L_{N}^{\left(j\right)}\right|\leq 5.0,\\ 0, & \mathrm{otherwise}. \end{array}\right.$$

To strike a balance between performance improvement and computational complexity, we employ a single-bit flipping strategy per trial in the studies presented in this paper. This approach ensures that the algorithm remains efficient while achieving significant performance gains.

The simplified D-SCF algorithm with the $M_{\alpha }\left(i\right)$ metric (3) serves as a performance benchmark for evaluating the effectiveness of the new algorithms introduced in the subsequent sections of this work. This comparison allows us to quantify the improvements and assess the trade-offs in decoding performance and complexity.

## 3. Th-SCF Decoder and Its Analysis

In this section, we propose the Th-SCF algorithm (Algorithm 1) in Section 3.1, followed by a comparative analysis with existing SCF algorithms in Section 3.2. In Section 3.3, we examine the distribution of the first error position (FEP) and compare the ability of various SCF algorithms to identify the true FEP. Section 3.4 provides a detailed comparison of the complexity across different SCF algorithms. Furthermore, we emphasize the key innovation of the proposed algorithm: it is built on a solid theoretical framework, which not only ensures performance comparable to previous threshold-based methods [10], but also offers the benefit of rigorous theoretical support for the flipping strategy. In Section 3.5, we prove that the Th-SCF algorithm delays the FEP with probability 1 (Theorem 1), providing a theoretical foundation for the performance improvements observed empirically.

For analytical tractability, we adopt the Gaussian approximation (GA) assumption, as provided in Section III of [18], which provides a valid approximation of the LLR distribution. Furthermore, we prove Proposition 1, which guarantees high average reliability for all information bits in the asymptotic regime.

Let $\mu_{N}^{\left(i\right)}$ denote the mean of the LLR $L_{N}^{\left(i\right)}$, with $\mu_{1}^{\left(1\right)}=\frac{2}{\sigma^{2}}$. The LLR sequence is defined as $L_{0:N}=\left[L_{N}^{\left(0\right)},\ldots ,L_{N}^{\left(N-1\right)}\right]$. We assume that all-zero codeword $0_{N}$ is transmitted as adopted in Section III of [18] with 0 representing an all-zero vector and adopt an information set A of size *K*, where i∈A satisfy $Z\left(W_{N}^{\left(i\right)}\right)<2^{-N^{\beta }}$ for $\beta \in (0,\frac{1}{2})$ as introduced in Proposition 18 of [1].

To begin with, we reformulate the GA assumption (see Section III of [18]) into a more convenient form, as summarized in Assumption 1. This assumption provides an effective and analytically tractable approximation of the LLR distribution for information bits, thereby facilitating the subsequent theoretical analysis.

**Assumption** **1.** *Assuming the LLRs of each subchannel follow a Gaussian distribution with mean equal to half the variance as introduced in Section III of [18] and assume that ${\hat{u}}_{0:i}=u_{0:i}$ (i.e., ${\hat{u}}_{0}=u_{0},\cdots ,{\hat{u}}_{i-1}=u_{i-1}$) when decoding $u_{i}$, then we have:*

$$\left\{L_{N}^{\left(i\right)}|{\hat{u}}_{0:i}=u_{0:i}\right\}∼N\left(\mu_{N}^{\left(i\right)},2\mu_{N}^{\left(i\right)}\right),$$

*where $\mu_{N}^{\left(i\right)}$ is recursively calculated by:*

$$\mu_{N}^{\left(i\right)}=\left\{\begin{array}{cc} \phi^{-1}\left(1-{\left(1-\phi \left(\mu_{N/2}^{\left(i/2\right)}\right)\right)}^{2}\right), & \mathrm{if}\;i\;\mathrm{is}\;\mathrm{even},\\ 2\mu_{N/2}^{(i-1)/2}, & \mathrm{if}\;i\;\mathrm{is}\;\mathrm{odd}, \end{array}\right.$$

*with ϕ(x) given by:*

$$\phi \left(x\right)≜1-\int_{-\infty }^{\infty }\frac{1}{\sqrt{4\pi x}}\tanh\left(\frac{t}{2}\right)e^{-\frac{{\left(t-x\right)}^{2}}{4x}}\;dt.$$

Proposition 1 supports the Th-SCF framework by ensuring asymptotically high average LLR magnitudes for all information bits. According to the statistical properties of the Gaussian distribution, bits with LLR magnitudes below $N^{\beta }$ are unreliable and likely correspond to the true FEP.

**Proposition** **1.** *For any i∈A and sufficiently large N, we obtain for any $\beta \in (0,\frac{1}{2})$ and any rate R<I(W) that:*

(4) $$\begin{array}{c} \mu_{N}^{\left(i\right)}\geq N^{\beta }. \end{array}$$

**Proof.**  Using Assumption 1 and the definition of the bit error rate $\mathrm{Pe}(W_{N}^{\left(i\right)})$ from Equation (17) of ref. [18], it follows from Proposition 18 of ref. [1] that ∀i∈A and any rate R<I(W), we have:

$$\mathrm{Pe}\left(W_{N}^{\left(i\right)}\right)=Q\left(\sqrt{\frac{\mu_{N}^{\left(i\right)}}{2}}\right)\leq Z\left(W_{N}^{\left(i\right)}\right)\leq 2^{-N^{\beta }},$$

which implies:

$$\sqrt{\frac{\mu_{N}^{\left(i\right)}}{2}}\geq Q^{-1}\left(2^{-N^{\beta }}\right).$$

To prove (4), it suffices to verify the following condition:

(5) $$\begin{array}{c} Q^{-1}\left(2^{-N^{\beta }}\right)\geq \sqrt{-\ln2^{-N^{\beta }}}=\sqrt{N^{\beta }\ln2}. \end{array}$$

This inequality shows that the LLR magnitude for the information bits grows asymptotically as $N^{\beta }$, guaranteeing high reliability for the information bits as *N* increases.

We now begin the analysis of inequality (5). Starting from the definition of the *Q*-function and applying the method of integration by parts, we obtain:

$$\frac{1}{x^{2}}\int_{x}^{+\infty }e^{-\frac{t^{2}}{2}}\;dt\geq \int_{x}^{+\infty }\frac{1}{t^{2}}e^{-\frac{t^{2}}{2}}\;dt=\frac{1}{x}e^{-\frac{x^{2}}{2}}-\int_{x}^{+\infty }e^{-\frac{t^{2}}{2}}\;dt.$$

This leads to:

$$\frac{1}{x}e^{-\frac{x^{2}}{2}}\leq \left(1+\frac{1}{x^{2}}\right)\int_{x}^{+\infty }e^{-\frac{t^{2}}{2}}\;dt,$$

which further implies the following lower bound on the *Q*-function:

$$Q\left(x\right)=\frac{1}{\sqrt{2\pi }}\int_{x}^{+\infty }e^{-\frac{t^{2}}{2}}\;dt\geq \frac{x}{\sqrt{2\pi }\left(1+x^{2}\right)}e^{-\frac{x^{2}}{2}}.$$

The above bound also appears in Equation (2.1.b) of ref. [19].

For sufficiently large *x*, this expression can be simplified as:

(6) $$Q\left(x\right)\geq \frac{x}{\sqrt{2\pi }\left(1+x^{2}\right)}e^{-\frac{x^{2}}{2}}\geq \frac{1}{x}e^{-\frac{x^{2}}{2}}=e^{-\frac{x^{2}}{2}-\ln x}\geq e^{-x^{2}},$$

where the last inequality follows from the fact that for sufficiently large *x*, we have $\frac{x^{2}}{2}\geq \ln x$.

Given that y=Q(x), we now invert the relationship to express *x* as $x=Q^{-1}\left(y\right)$. For sufficiently small *y*, which implies that *x* is sufficiently large, we have the following inequality:

$$-\ln y\leq x^{2}={\left(Q^{-1}\left(y\right)\right)}^{2}.$$

Therefore, for sufficiently small *y*, we can conclude:

$$Q^{-1}\left(y\right)\geq \sqrt{-\ln y}.$$

Substituting $y=2^{-N^{\beta }}$, we obtain, for sufficiently large *N*:

$$Q^{-1}\left(2^{-N^{\beta }}\right)\geq \sqrt{-\ln2^{-N^{\beta }}}=\sqrt{N^{\beta }\ln2}.$$

Hence, we derive that for any i∈A and sufficiently large *N*, the following holds:

$$\begin{array}{c} \sqrt{\frac{\mu_{N}^{\left(i\right)}}{2}}\geq Q^{-1}\left(2^{-N^{\beta }}\right)\geq \sqrt{N^{\beta }\ln2}, \end{array}$$

which implies that

$$\begin{array}{c} \mu_{N}^{\left(i\right)}\geq \left(2\ln2\right)N^{\beta }>N^{\beta }. \end{array}$$

Thus, the result is established. □

Proposition 1 serves as the foundation for our subsequent analysis by highlighting a key asymptotic property: the LLRs of all information bits in a polar code become increasingly large, indicating high reliability as the block length grows. This insight allows us to establish a practical criterion—if the LLR magnitude of a particular information bit falls below $N^{\beta }$, it can be considered unreliable in the asymptotic sense. Consequently, such a bit is more likely to correspond to the true location of a first error position (FEP), and flipping its hard decision result has the potential to significantly enhance decoding performance.

### 3.1. Proposed Th-SCF Decoding Algorithm

This section presents the Threshold SCF (Th-SCF) algorithm (Algorithm 1), which leverages the result from Proposition 1, ensuring asymptotically high reliability for all information bits i∈A.

In Algorithm 1, the function $\mathrm{SCDecoder}(y_{0:N},A,k)$ executes the standard successive cancellation (SC) decoding process. When k=0, it performs regular SC decoding without modification. When k>0, the decoder proceeds as usual but intentionally flips the decoding result of the information bit $u_{k}$, simulating a correction at a potentially erroneous position.
**Algorithm 1** Th-SCF Decoding Algorithm**Input:** Original received symbols $y_{0:N}$, information set A, the maximum attempts *T*, predefined threshold $T_{f}$
**Output:** The decoded codewords ${\hat{u}}_{0:N}$
  1:(${\hat{u}}_{0:N}$,$L_{0:N}$) ←SCDecoder($y_{0:N}$,A,0);  2:**if** CRCChek$({\hat{u}}_{0:N})=\mathrm{fail}$ **then**  3:     $S\leftarrow \mathrm{FlipSelector}(L_{0:N},T_{f},A)$;  4:     **for** t=1 to *T* **do**  5:          ${\hat{u}}_{0:N}$←SCDecoder($y_{0:N}$,A,$i_{t}$);  6:          **if** CRCChek$({\hat{u}}_{0:N})=\mathrm{success}$ **then** return ${\hat{u}}_{0:N}$;  7:                break  8:          **end if**  9:     **end for**10:**end if**


The function $\mathrm{FlipSelector}(L_{0:N},T_{f},A)$ is responsible for identifying a candidate set of error-prone bits based on their reliability metrics. Specifically, it selects the first *T* indices $S=\{i_{1}<i_{2}<\cdots <i_{T}\}\subseteq A$ such that the magnitude of the LLR $|L_{i_{t}}|$ for each index is less than or equal to a predefined threshold $T_{f}$. This threshold is chosen to satisfy the condition $T_{f}\leq CN^{\beta }$, where C∈(0,1) is a constant and $\beta \in (0,\frac{1}{2})$ is an empirically optimized parameter, typically determined through offline Monte Carlo simulations. By quantifying bit reliability in this way, the algorithm effectively isolates those information bits that are most susceptible to decoding errors, thereby guiding the SC Flip decoding process toward more promising correction candidates.

### 3.2. Comparative Analysis with Existing SCF Algorithms

In this section, we provide an in-depth analysis of the threshold SCF (Th-SCF) algorithm (Algorithm 1) and compare it with the min-LLR SCF algorithm (see Figure 5 of ref. [6]) simplified D-SCF algorithm [17] and the improved SCF algorithm as proposed in Section III of ref. [10].

The min-LLR SCF algorithm selects the *T* candidate indices with the smallest LLR magnitudes as described in Figure 5 of ref. [6], but it often struggles to accurately identify the true first error position (FEP) bits due to the sequential nature of the SC decoding process as observed in Section III of ref. [10]. The simplified D-SCF algorithm [17] enhances the min-LLR SCF algorithm by incorporating both the reliability of the bits and their positions in the information set A. This improvement leads to a significant performance boost while keeping the computational complexity on par with the min-LLR SCF algorithm [17]. Specifically, this algorithm selects the bit indices corresponding to the *T* smallest values of $M_{\alpha }\left(i\right)$, as defined in (3), to construct the set of bits to flip.

Compared to the min-LLR SCF and simplified D-SCF decoding algorithms, the Th-SCF algorithm offers a more straightforward approach to determining the bit positions that need to be flipped. As outlined in Algorithm 1, the core idea of the Th-SCF algorithm can be summarized as follows: Proposition 1 shows that the average LLR magnitudes of all information bits are greater than $N^{\beta }$. Based on this insight, we define a threshold criterion: when the LLR magnitude of a bit falls below a predefined threshold $T_{f}$, it reliably signals that this bit is unreliable and should be flipped. This approach eliminates the need for complex sorting operations, which are typically computationally expensive in traditional SCF methods.

The key contribution of our approach, compared to existing SCF decoding algorithms, lies in the rigorous theoretical framework underpinning the Th-SCF algorithm. While similar threshold-based techniques were proposed in [10], they mainly relied on experimental observations to select flipped bits and set the flipping threshold. In contrast, we rigorously prove that our algorithm can asymptotically identify the first error position (FEP) with probability 1 after a single threshold flip, providing a robust theoretical guarantee that was previously missing. This solid theoretical foundation significantly enhances the potential for improving SC decoding performance.

The critical set SCF algorithm [7] is another approach that determines the critical set offline and stores it for future use. While effective, this method increases storage requirements. In contrast, our threshold-based SCF algorithm selects the flip set by comparing the LLR magnitude of each information bit to a predefined threshold $T_{f}$. A significant advantage of our approach lies in its rigorous theoretical foundation, which provides a clear explanation for its performance improvements. Unlike the critical set method [7] and other experimental flip-based approaches [10], our algorithm not only enhances SC decoding performance but also offers valuable insights into designing more effective decoding strategies backed by solid theoretical guarantees.

For the first time, we provide a rigorous analysis of the LLR magnitude difference between correctly and incorrectly decoded bits. As demonstrated in Proposition 1, asymptotically, the average LLR magnitude of each information bit is high. Under the Gaussian approximation (GA) assumption (Assumption 1), the LLR of each information bit follows a Gaussian distribution, where the mean is equal to half the variance. This insight leads us to conclude that, when decoding errors occur, the LLR magnitudes of the erroneous bits are small, which is consistent with the observations in previous work (see Figure 10 of ref. [10]).

### 3.3. FEP Distribution and the Capability of SCF Algorithms to Identify the True FEP

To more clearly demonstrate the advantages of our proposed algorithm over existing SCF algorithms, such as the min-LLR SCF algorithm (Figure 5 of ref. [6]), we have plotted the distribution of the first error position (FEP) in the SC decoder across different signal-to-noise ratio (SNR). We also compare the probability of correctly identifying the FEP for both the proposed algorithm and the min-LLR SCF algorithm, with the maximum number of flips limited to T=10. This comparison further reinforces the benefits of our proposed algorithm.

As an example, we consider a (1024,512+12) polar code with the check polynomial $g\left(x\right)=x^{13}+x^{11}+x^{3}+x^{2}+1$. The information bits are generated using the GA method, with the design SNR set to 2.5 dB [18]. We then statistically analyze the probability distribution of the first error position (FEP) of the SC decoder across different SNR values, the decoding process continues until 400 errors are detected at each SNR setting.

**Figure 2:**
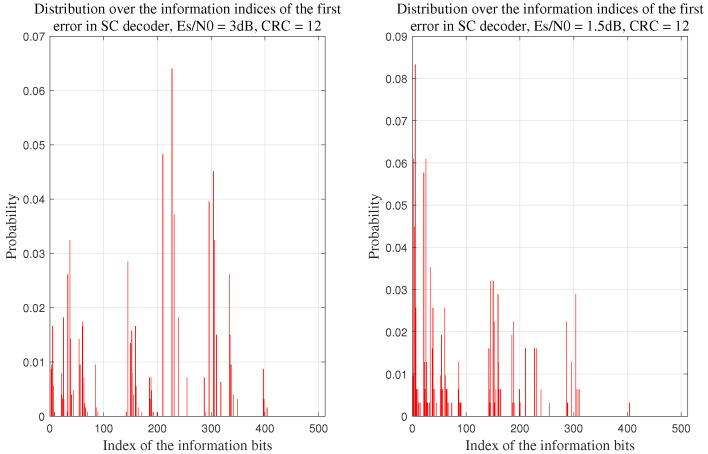
Distribution of the first error position (FEP) in the SC decoder across codeword indices at different SNRs with N=1024, $R=\frac{1}{2}$ and 12 bit CRC.

The results from Figure 2 indicate that as the SNR increases, the distribution of the FEP across the information set A becomes more uniform, a trend also observed in [[10], Figure 13], making it more difficult to accurately identify the FEP.

To better highlight the superiority of the proposed algorithm, we compare its ability to identify the true FEP with that of the min-LLR SCF algorithm across different code lengths and SNRs. A higher probability of correctly identifying the true FEP signifies a greater potential for performance improvement.

To gain a clearer understanding of the ability of different SCF algorithms to identify the true FEP, we formally define the flip sets generated by each SCF algorithm, as well as the corresponding probabilities of correctly identifying the true FEP.

For the min-LLR SCF algorithm, the flip set $F_{\min-\mathrm{LLR}}$ is the set of indices i∈A corresponding to the *T* smallest LLR magnitudes $|L_{N}^{\left(i\right)}|$, formally defined as:

(7) $$F_{\min-\mathrm{LLR}}=\left\{i_{1},\ldots ,i_{T}||L_{N}^{\left(i_{1}\right)}\left|\leq \cdots \leq \right|L_{N}^{\left(i_{T}\right)}\left|\leq \right|L_{N}^{\left(j\right)}|,\;\forall j\in A\setminus \left\{i_{1},\ldots ,i_{T}\right\}\right\}.$$

For the proposed Th-SCF algorithm (Algorithm 1), the flip set $F_{\mathrm{Th}-\mathrm{SCF}}$ consists of the first *T* indices $\{i_{1}<i_{2}<\cdots <i_{T}\}\subset A$, such that for all k∈{1,2,…,T}, the condition $|L_{N}^{\left(i_{k}\right)}|\leq T_{f}$ is satisfied. This can be expressed as:

(8) $$F_{\mathrm{Th}-\mathrm{SCF}}=\{i_{1},i_{2},\ldots ,i_{T}|i_{k}\in A,\;\mathrm{for}\;\mathrm{the}\;\mathrm{first}\;T\;\mathrm{indices}\;\mathrm{satisfying}\;|L_{N}^{\left(i_{k}\right)}\left|\leq T_{f}\right\}.$$

In these definitions, A represents the information set, and $T_{f}$ denotes the predefined threshold used in the Th-SCF algorithm. The main distinction between the two algorithms lies in their selection criteria: while the min-LLR SCF algorithm chooses the indices corresponding to the *T* smallest LLR magnitudes, the Th-SCF algorithm selects the first *T* indices for which the LLR magnitudes fall below a predefined threshold $T_{f}$.

We define the effectiveness of different SCF algorithms in identifying the true first error position (FEP) as the probability that the true FEP is included in the flip set. This can be formally expressed as:

(9) $$P_{\mathrm{find}}=P\left({\mathrm{idx}}_{\mathrm{FEP}}\in F_{\mathrm{SCF}}\right),$$

where $F_{\mathrm{SCF}}$ represents the flip set generated by the SCF algorithm, either $F_{\min-\mathrm{LLR}}$ from Equation (7) or $F_{\mathrm{Th}-\mathrm{SCF}}$ from Equation (8), and ${\mathrm{idx}}_{\mathrm{FEP}}$ refers to the index of the true first error position in the SC decoder. This probability quantifies the effectiveness of the SCF algorithm in successfully identifying the true FEP after the flipping process.

To better highlight the advantages of the proposed algorithm, we focus on two code lengths, N=1024 and N=4096, with a CRC length of 12 and a check polynomial $g\left(x\right)=x^{13}+x^{11}+x^{3}+x^{2}+1$, and a code rate of $\frac{1}{2}$, while the maximum flipping attempts $T_{\max}=10$. The information set A is generated using the GA method [18] and the design SNR for each code length is selected to ensure that the SC decoder achieves optimal performance when the block error rate (BLER) is $10^{-2}$, as recommended in [6]. Specifically, the design SNRs for the GA method are set to 2.5 dB for N=1024 and 2.15 dB for N=4096.

**Figure 3:**
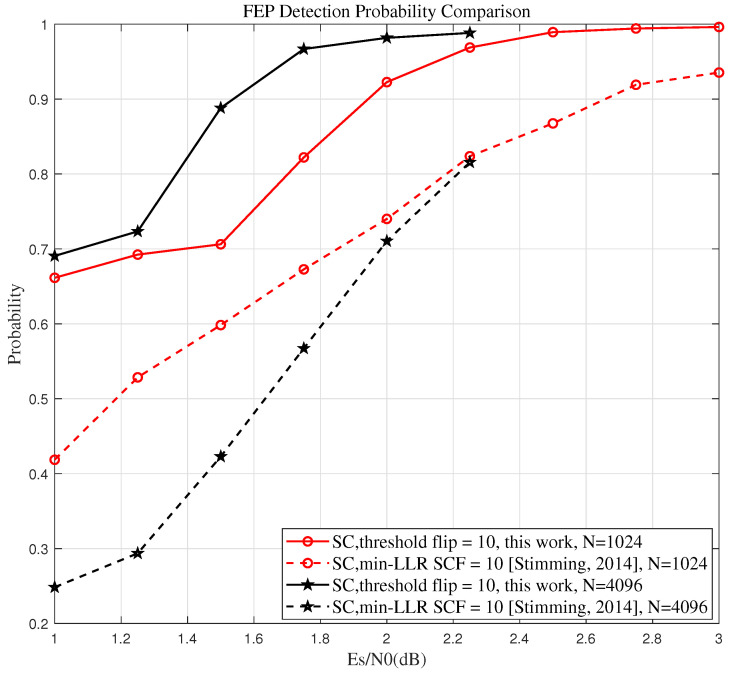
Probability of correctly identifying the true FEP by the proposed algorithm and the min-LLR SCF algorithm across different code lengths andSNRs ([6]).

Figure 3 visualizes the probability defined in Equation (9). The two solid lines illustrate the variation in the probability of the Th-SCF algorithm correctly identifying the true FEP with SNR for different code lengths, whereas the two dashed lines represent the corresponding probabilities for the min-LLR SCF algorithm.

From Figure 3, we can observe that, for various code lengths and SNRs, the proposed Th-SCF algorithm outperforms the min-LLR SCF algorithm in terms of identifying the true FEP. Moreover, as the code length *N* increases, the advantage of the proposed algorithm over the min-LLR SCF algorithm becomes more significant. This trend helps explain the improved performance observed in the experimental results.

### 3.4. Complexity Analysis of the Proposed Th-SCF Algorithm and the Existing SCF Algorithms

In this section, we derive the worst-case and average-case complexities of the SCF algorithm [6], simplified D-SCF algorithm [17], as well as the proposed Th-SCF algorithm.

To provide a clearer understanding of how worst-case and average complexities are calculated, we restate the conclusions from Propositions 1 and 2 of ref. [6] as follows:

**Proposition** **2** (Worst-case complexity of the min-LLR SCF algorithm)**.** *The worst-case computational complexity of the min-LLR SCF algorithm (see Figure 5 of ref. [6]) is $O(TN\log_{2}N)$, where T represents the maximum number of flipping attempts.*

**Proposition** **3** (Average complexity of the min-LLR SCF algorithm)**.** *Let $P_{e}\left(R,\mathrm{SNR}\right)$ denote the block error rate of a polar code of rate R at a given SNR. The average computational complexity of the min-LLR SCF algorithm is $O\left(N\log_{2}N\left(1+T\cdot P_{e}\left(R,\mathrm{SNR}\right)\right)\right)$, where $R=\frac{K+r}{N}$, with r being the CRC length.*

As noted in [17], the performance of the simplified D-SCF algorithm is essentially comparable to that of the D-SCF algorithm in various scenarios. Therefore, we use the simplified D-SCF algorithm as the focus of our subsequent experiments. Since the computation method of the simplified D-SCF algorithm closely matches that of the min-LLR SCF algorithm [17], their computational complexities are almost the same. Moreover, based on the operational principles of the Th-SCF algorithm (Algorithm 1), we observe that its worst-case complexity is also similar to that of the min-LLR SCF algorithm.

Although the proposed algorithm does not exhibit a significant advantage in worst-case complexity compared to the min-LLR SCF and simplified D-SCF algorithms, it demonstrates notable improvements in terms of average complexity. Specifically, we find that the average computational complexity of the SCF algorithm is closely related to the average number of iterations. For both the min-LLR SCF and simplified D-SCF algorithms, each iteration involves SC decoding, CRC decoding, and an additional step of selecting and sorting the flipping indices based on a specific metric, such as the LLR magnitudes or the metric $M_{\alpha }\left(i\right)$, as defined in Equation (3), when CRC decoding fails. In contrast, an iteration of the proposed Th-SCF algorithm consists of SC decoding, CRC decoding, and a simpler comparison step to check if the LLR magnitude falls below the predefined threshold $T_{f}$. This simplified procedure reduces the computational complexity, making the proposed algorithm more efficient in terms of average performance.

As a result, the complexity of our Th-SCF algorithm, which encompasses the computational and sorting complexities, is lower than that of existing SCF algorithms. It is worth noting that previous studies have shown that threshold-based strategies can achieve lower average complexity compared to min-LLR SCF decoders as observed in Figures 19 and 20 of ref. [10]. A more detailed comparison of the complexity of various SCF algorithms will be provided in Section 4.2, offering a more intuitive illustration of the advantages of our proposed algorithm over other flip approaches.

It should also be noted that the flipping method provided in [10] shares similarities with our approach. However, our proposed Th-SCF decoding algorithm is distinguished by its rigorous theoretical foundation, as established in Proposition 1 and Theorem 1. In Section 3.5, we further demonstrate that, in the asymptotic case, a single threshold flip can identify the true first error position (FEP) of the SC decoder with probability 1, thereby improving the performance of the decoder with high probability. This marks the first theoretical guarantee for the effectiveness of an SCF decoding algorithm, providing a solid and principled basis for its practical deployment.

### 3.5. Theoretical Analysis of the Th-SCF Algorithm

In this section, we analyze the probability of delaying the first error position (FEP) after one threshold flip in the Th-SCF algorithm. Our analysis shows that, asymptotically, the FEP is delayed with probability 1 (Theorem 1), thus paving the way for the development of more efficient flip algorithms.

Let ${\hat{u}}_{0:N}^{\left(0\right)}$ and ${\hat{u}}_{0:N}^{\left(1\right)}$ denote the SC and Th-SCF decoding results, respectively. Let $\tau_{0}$ and $\tau_{1}$ represent their corresponding FEPs. We define the error events as follows:

$$\begin{array}{c} \left\{\tau_{0}=i\right\}=\left\{{\hat{u}}_{i}^{\left(0\right)}=1,{\hat{u}}_{0:i}^{\left(0\right)}=0\right\},\\ \left\{\tau_{1}=i\right\}=\left\{{\hat{u}}_{i}^{\left(1\right)}=1,{\hat{u}}_{0:i}^{\left(1\right)}=0\right\}. \end{array}$$

This section focuses on the asymptotic probabilities of the FEP being delayed (10), unchanged (11), or advanced (12) following an SC failure event $\left\{\tau_{0}<N\right\}$. Specifically, we are interested in the following probabilities:

(10) $$P(\tau_{1}>\tau_{0}|\tau_{0}<N),$$

(11) $$P(\tau_{1}=\tau_{0}|\tau_{0}<N),$$

(12) $$P(\tau_{1}<\tau_{0}|\tau_{0}<N).$$

The events in question correspond to the scenarios where the FEP is delayed, unchanged, or advanced after a threshold flip, respectively.

By applying the law of total probability, we derive the following for $T_{f}\leq CN^{\beta }$ (with 0<C<1 a constant) and any Borel set *B*, where i,j∈A and j<i:

$$\begin{array}{cc} & P\left(L_{N}^{\left(i\right)}\in B|L_{0:i}>0\right)=P\left(L_{N}^{\left(i\right)}\in B,L_{N}^{\left(j\right)}>T_{f}|L_{0:i}>0\right)+P\left(L_{N}^{\left(i\right)}\in B,0<L_{N}^{\left(j\right)}\leq T_{f}|L_{0:i}>0\right), \end{array}$$

where $L_{0:i}>0$ indicates that $L_{N}^{\left(0\right)}>0,\cdots ,L_{N}^{\left(i-1\right)}>0$.

By splitting this probability into two cases, one where $L_{N}^{\left(j\right)}\geq T_{f}$ and the other where $L_{N}^{\left(j\right)}\leq T_{f}$, we can then apply the conditional probability formula to derive the following result:

$$\begin{array}{cc} & P(L_{N}^{\left(i\right)}\in B|L_{0:i}>0)\\ = & P\left(L_{N}^{\left(i\right)}\in B|L_{0:i}>0,L_{N}^{\left(j\right)}>T_{f}\right)P\left(L_{N}^{\left(j\right)}>T_{f}|L_{0:i}>0\right)\\ + & P\left(L_{N}^{\left(i\right)}\in B|L_{0:i}>0,0<L_{N}^{\left(j\right)}\leq T_{f}\right)P\left(0<L_{N}^{\left(j\right)}\leq T_{f}|L_{0:i}>0\right)\\ \leq & P\left(L_{N}^{\left(i\right)}\in B|L_{0:i}>0,L_{N}^{\left(j\right)}>T_{f}\right)+P\left(0<L_{N}^{\left(j\right)}\leq T_{f}|L_{0:i}>0\right), \end{array}$$

Similarly, we can derive a lower bound:

$$\begin{array}{cc} & P\left(L_{N}^{\left(i\right)}\in B|L_{0:i}>0\right)\geq P\left(L_{N}^{\left(i\right)}\in B,L_{N}^{\left(j\right)}>T_{f}|L_{0:i}>0\right)\\ = & P\left(L_{N}^{\left(i\right)}\in B|L_{0:i}>0,L_{N}^{\left(j\right)}>T_{f}\right)P\left(L_{N}^{\left(j\right)}>T_{f}|L_{0:i}>0\right)\\ = & P\left(L_{N}^{\left(i\right)}\in B|L_{0:i}>0,L_{N}^{\left(j\right)}>T_{f}\right)\left(1-P\left(0<L_{N}^{\left(j\right)}\leq T_{f}|L_{0:i}>0\right)\right)\\ = & P(L_{N}^{\left(i\right)}\in B|L_{0:i}>0,L_{N}^{\left(j\right)}>T_{f})\\ - & P\left(L_{N}^{\left(i\right)}\in B|L_{0:i}>0,L_{N}^{\left(j\right)}>T_{f}\right)P\left(0<L_{N}^{\left(j\right)}\leq T_{f}|L_{0:i}>0\right)]\\ \geq & P\left(L_{N}^{\left(i\right)}\in B|L_{0:i}>0,L_{N}^{\left(j\right)}>T_{f}\right)-P\left(0<L_{N}^{\left(j\right)}\leq T_{f}|L_{0:i}>0\right). \end{array}$$

Thus, we obtain for sufficiently large *N* and any Borel set *B* with $T_{f}\leq CN^{\beta }$ that:

$$\begin{array}{cc} & \left|P\left(L_{N}^{\left(i\right)}\in B|L_{0:i}>0\right)-P\left(L_{N}^{\left(i\right)}\in B|L_{0:i}>0,L_{N}^{\left(j\right)}>T_{f}\right)\right|\\ \leq & P(0<L_{N}^{\left(j\right)}\leq T_{f}|L_{0:i}>0)\\ \leq & P(L_{N}^{\left(j\right)}\leq T_{f}|L_{0:i}>0)\\ = & \Phi \left(\frac{T_{f}-\mu_{N}^{\left(j\right)}}{\sqrt{2\mu_{N}^{\left(j\right)}}}\right)=Q\left(\frac{\mu_{N}^{\left(j\right)}-T_{f}}{\sqrt{2\mu_{N}^{\left(j\right)}}}\right)\\ \overset{\left(a\right)}{\leq } & Q(\frac{\left(1-C\right)N^{\frac{\beta }{2}}}{\sqrt{2}})\to 0, \end{array}$$

where

$$\frac{\mu_{N}^{\left(j\right)}-T_{f}}{\sqrt{2\mu_{N}^{\left(j\right)}}}=\sqrt{\frac{\mu_{N}^{\left(j\right)}}{2}}-\frac{T_{f}}{\sqrt{2\mu_{N}^{\left(j\right)}}}\geq \frac{N^{\frac{\beta }{2}}}{\sqrt{2}}-\frac{CN^{\frac{\beta }{2}}}{\sqrt{2}},$$

and the condition $T_{f}\leq CN^{\beta }$, together with Proposition 1, leads to (a).

This allows us to conclude that for any i,j∈A with j<i and any Borel set *B*, for sufficiently large *N* and any $T_{f}\leq CN^{\beta }$ with 0<C<1, the following result holds:

(13) $$P\left(L_{N}^{\left(i\right)}\in B|L_{0:i}>0,L_{N}^{\left(j\right)}>T_{f}\right)\approx P\left(L_{N}^{\left(i\right)}\in B|L_{0:i}>0\right).$$

This approximation simplifies our analysis, as it implies that when *N* is sufficiently large and the threshold condition $T_{f}\leq CN^{\beta }$ are met, the correctly decoded preceding bits have a negligible effect on the decoding probability of the current bit.

The main result of this paper is summarized in the following theorem, which shows that, asymptotically, the Th-SCF algorithm delays the FEP with probability 1, thereby improving the performance of the SC decoder.

**Theorem** **1.** *For sufficiently large N, some $\beta \in (0,\frac{1}{2})$, we obtain for any $T_{f}\leq CN^{\beta }$ with 0<C<1 that:*

(14) $$\lim_{N\to \infty }P\left(\tau_{1}>\tau_{0}|\tau_{0}<N\right)=1.$$

**Proof.**  To improve the clarity and structure of our proof, we define two types of errors: Type-I error and Type-II error. A Type-I error occurs when bit *i* is not flipped despite the FEP is at *i*. In contrast, a Type-II error occurs when there exist a j∈A with j<i is incorrectly flipped if the FEP is at *i*.

Under the hard-decision framework of SC decoding, we establish a relationship between the FEP $\tau_{0}$ and the LLRs at the hard-decision side ($L_{N}^{\left(i\right)}$ for all i∈A), allowing for efficient error localization without the need for exhaustive search.

$$\begin{array}{c} \left\{\tau_{0}=i\right\}=\left\{{\hat{u}}_{i}^{\left(0\right)}=1,{\hat{u}}_{0:i}^{\left(0\right)}=0\right\}=\left\{L_{N}^{\left(i\right)}<0,L_{0:i}>0\right\}. \end{array}$$

Let *X* denote the index of the first bit that is flipped. To evaluate the delay probability of the first error position (FEP), denoted as *i*, after a threshold flip, we only need to compute the following probability:

$$\begin{array}{cc} & P(\tau_{1}\leq \tau_{0}|\tau_{0}<N)\\ = & \frac{P(\tau_{1}\leq \tau_{0},\tau_{0}<N)}{P(\tau_{0}<N)}\\ = & \frac{\sum_{i\in A}P\left(\tau_{1}\leq \tau_{0},\tau_{0}=i\right)}{P(\tau_{0}<N)}\\ = & \frac{\sum_{i\in A}P\left(\tau_{1}\leq \tau_{0}|\tau_{0}=i\right)P\left(\tau_{0}=i\right)}{P(\tau_{0}<N)}\\ \overset{\left(i\right)}{=} & \frac{\sum_{i\in A}P\left(X\neq i|\tau_{0}=i\right)P\left(\tau_{0}=i\right)}{P(\tau_{0}<N)}, \end{array}$$

where

$$\begin{array}{c} \begin{array}{cc} & P(X\neq i|\tau_{0}=i)\\ = & P(\{\exists j<i,j\in A,s.t.,\;|L_{N}^{\left(j\right)}|<T_{f}\}\cup \{|L_{N}^{\left(i\right)}|\geq T_{f}\}|\tau_{0}=i)\\ \leq & \sum_{j\in A,j<i}\underset{\mathrm{Type}-\mathrm{II}\;\mathrm{error}}{P\left(|L_{N}^{\left(j\right)}|<T_{f}|\tau_{0}=i\right)}+\underset{\mathrm{Type}-I\;\mathrm{error}}{P\left(|L_{N}^{\left(i\right)}|\geq T_{f}|\tau_{0}=i\right)}, \end{array} \end{array}$$

with the last inequality follows from the union bound, and (i) holds because when the FEP is at bit $u_{i}$ (i.e., $\tau_{0}=i$), and the index of the first flipped bit is also *i* (or equivalently, X=i), the FEP must be delayed after performing one threshold flip.

If there exists a j∈A with j<i such that $|L_{N}^{\left(j\right)}|<T_{f}$, which corresponds to a Type-II error, then, by the law of total probability, we decompose $L_{0:i}$ into $L_{0:j}$, $L_{N}^{\left(j\right)}$, and $L_{j+1:i}$, resulting in:

(15) $$\begin{array}{cc} & P\left(|L_{N}^{\left(j\right)}|<T_{f}|\tau_{0}=i\right)=1-P\left(L_{N}^{\left(j\right)}>T_{f}|\tau_{0}=i\right)\\ = & 1-\frac{P(L_{N}^{\left(j\right)}>T_{f},L_{0:i}>0,L_{N}^{\left(i\right)}<0)}{P(L_{0:i}>0,L_{N}^{\left(i\right)}<0)}\\ \overset{\left(b\right)}{=} & 1-\frac{P\left(L_{N}^{\left(j\right)}>T_{f}|L_{0:j}>0\right)P\left(L_{N}^{\left(i\right)}<0|L_{0:i}>0,L_{N}^{\left(j\right)}>T_{f}\right)}{P\left(L_{N}^{\left(j\right)}>0|L_{0:j}>0\right)P\left(L_{N}^{\left(i\right)}<0|L_{0:i}>0\right)}\\ \times & \frac{\prod_{k=j+1}^{i-1}P\left(L_{k}>0|L_{0:k}>0,L_{N}^{\left(j\right)}>T_{f}\right)}{\prod_{k=j+1}^{i-1}P\left(L_{k}>0|L_{0:k}>0\right)}\\ \overset{\left(c\right)}{\approx } & 1-\frac{P(L_{N}^{\left(j\right)}>T_{f}|L_{0:j}>0)}{P(L_{N}^{\left(j\right)}>0|L_{0:j}>0)}\leq 1-P\left(L_{N}^{\left(j\right)}>T_{f}|L_{0:j}>0\right)\\ = & 1-Q\left(\frac{T_{f}-\mu_{N}^{\left(j\right)}}{\sqrt{2\mu_{N}^{\left(j\right)}}}\right)=Q\left(\frac{\mu_{N}^{\left(j\right)}-T_{f}}{\sqrt{2\mu_{N}^{\left(j\right)}}}\right). \end{array}$$

where (b) follows from the fact that

$$P\left(L_{N}^{\left(k\right)}>0|L_{0:k}>0,L_{N}^{\left(j\right)}>T_{f}\right)=P\left(L_{N}^{\left(k\right)}>0|L_{0:k}>0\right)$$

for any k,j∈A with k<j and (13) yields (c).

In situations where no $L_{N}^{\left(i\right)}$ meets the threshold flip criterion or a Type-I error occurs, it follows that:

(16) $$\begin{array}{cc} & P(|L_{N}^{\left(i\right)}|\geq T_{f}|\tau_{0}=i)\\ = & \frac{P(L_{0:i}>0,L_{N}^{\left(i\right)}<0,L_{N}^{\left(i\right)}\leq -T_{f})}{P(L_{0:i}>0,L_{N}^{\left(i\right)}<0)}\\ = & \frac{P\left(L_{N}^{\left(i\right)}\leq -T_{f}|L_{0:i}>0\right)P\left(L_{0:i}>0\right)}{P\left(L_{N}^{\left(i\right)}<0|L_{0:i}>0\right)P\left(L_{0:i}>0\right)}\\ = & \frac{\Phi (-\frac{\mu_{N}^{\left(i\right)}+T_{f}}{\sqrt{2\mu_{N}^{\left(i\right)}}})}{\Phi (-\sqrt{\frac{\mu_{N}^{\left(i\right)}}{2}})}\\ = & \frac{Q(\frac{\mu_{N}^{\left(i\right)}+T_{f}}{\sqrt{2\mu_{N}^{\left(i\right)}}})}{Q(\sqrt{\frac{\mu_{N}^{\left(i\right)}}{2}})}. \end{array}$$

Noting that $T_{f}\leq CN^{\beta }$ and applying Proposition 1, we obtain for all i,j∈A with j<i that:

$$\frac{\mu_{N}^{\left(j\right)}-T_{f}}{\sqrt{2\mu_{N}^{\left(j\right)}}}=\sqrt{\frac{\mu_{N}^{\left(j\right)}}{2}}-\frac{T_{f}}{\sqrt{2\mu_{N}^{\left(j\right)}}}\geq \frac{N^{\frac{\beta }{2}}}{\sqrt{2}}-\frac{CN^{\frac{\beta }{2}}}{\sqrt{2}}.$$

By combining the bounds previously established in Equations (15) and (16), we conclude that, there exists a constant 0<b<β such that the following result holds:

$$\begin{array}{cc} & P(X\neq i|\tau_{0}=i)\\ \leq & \sum_{j\in A,j<i}\underset{\mathrm{Type}-\mathrm{II}\;\mathrm{error}}{P\left(|L_{N}^{\left(j\right)}|<T_{f}|\tau_{0}=i\right)}+\underset{\mathrm{Type}-I\;\mathrm{error}}{P\left(|L_{N}^{\left(i\right)}|\geq T_{f}|\tau_{0}=i\right)}\\ \leq & NQ\left(\frac{\mu_{N}^{\left(j\right)}-T_{f}}{\sqrt{2\mu_{N}^{\left(j\right)}}}\right)+\frac{Q(\frac{\mu_{N}^{\left(i\right)}+T_{f}}{\sqrt{2\mu_{N}^{\left(i\right)}}})}{Q(\sqrt{\frac{\mu_{N}^{\left(i\right)}}{2}})}\\ \overset{\left(d\right)}{\leq } & NQ\left(\frac{N^{\beta }-CN^{\beta }}{\sqrt{2N^{\beta }}}\right)+\frac{e^{-\frac{{\left(\mu_{N}^{\left(i\right)}+T_{f}\right)}^{2}}{4\mu_{N}^{\left(i\right)}}}}{e^{-\frac{\mu_{N}^{\left(i\right)}}{4}}}\\ = & NQ\left(\frac{N^{\beta }-CN^{\beta }}{\sqrt{2N^{\beta }}}\right)+e^{-\frac{T_{f}}{2}-\frac{T_{f}^{2}}{4\mu_{N}^{\left(i\right)}}}\\ \overset{\left(e\right)}{\leq } & Ne^{-\frac{{\left(1-C\right)}^{2}}{4}N^{\beta }}+e^{-\left(C+C^{2}\right)N^{\beta }}\\ \leq & e^{-N^{b}}, \end{array}$$

where the approximation $Q\left(x\right)\approx \frac{1}{2}\exp\left(-\frac{x^{2}}{2}\right)$, valid for sufficiently large *x* from Equation (9) of ref. [20], justifies step (d). Step (e) then follows from the assumption that for all i∈A and some constant 0<C<1, we have:

$$\begin{array}{c} T_{f}\leq CN^{\beta },\;\frac{T_{f}^{2}}{\mu_{N}^{\left(i\right)}}\leq \frac{C^{2}N^{2\beta }}{N^{\beta }}=C^{2}N^{\beta }. \end{array}$$

With the use of the conditional probability formula, we can infer that for some 0<b<β and sufficiently large *N*:

$$\begin{array}{cc} P(X\neq \tau_{0}|\tau_{0}<N)= & \frac{\sum_{i\in A}P\left(X\neq i|\tau_{0}=i\right)P\left(\tau_{0}=i\right)}{P(\tau_{0}<N)}\\ \leq & e^{-N^{b}}\frac{\sum_{i\in A}P\left(\tau_{0}=i\right)}{P(\tau_{0}<N)}=e^{-N^{b}}, \end{array}$$

which implies that the Th-SCF algorithm flips the true FEP with probability 1, thus delaying the FEP with probability 1!

Accordingly, the proof of Theorem 1 is established. □

Theorem 1 confirms that Th-SCF algorithm delays the FEP with probability 1, ensuring improved SC decoder performance. This provides a strong theoretical foundation for designing effective SCF strategies tailored for the SC decoder.

## 4. Performance Analysis of the Th-SCF Algorithm

This section presents simulation results for a binary-input additive white Gaussian noise (BI-AWGN) channel, where the information bit set A is generated using the Gaussian approximation (GA) method [18] to ensure that the SNR required for the SC algorithm to achieve a BLER of $10^{-2}$ is minimized for different code lengths. Decoding continues until 400 errors are detected for each code length and code rate. To evaluate the performance of the proposed algorithm, we use a 12-bit CRC as suggested in [10] for all SCF decoding algorithms, including the min-LLR SCF algorithm [6], simplified D-SCF algorithm [17], improved SCF algorithm [10], and the proposed Th-SCF algorithm, with the check polynomial $g\left(x\right)=x^{13}+x^{11}+x^{3}+x^{2}+1$. For the SCL decoding algorithm, we use a CRC length of 8 bits, as recommended in [10], with the check polynomial $g\left(x\right)=x^{8}+x^{6}+x^{3}+x^{2}+1$.

To assess the upper limit of error correction for the proposed algorithm, we also compared the BLER for SC Oracle-1 (SCO-1) decoding as proposed in Section III-C of ref. [6] using a 12 bit CRC, which is only allowed to intervene only once during the decoding process to correct the first erroneous bit decision (i.e., the first error position, or FEP, as discussed earlier), as suggested in Section IV of [10].

To enhance the practicality of the proposed algorithm, the threshold $T_{f}$ is determined through a two-step process:

(a). It is computed through offline Monte Carlo simulations to ensure the Th-SCF algorithm achieves at least 75% reliability in identifying the true FEP, i.e.,
  (17) $$P(i\in S|\tau_{0}=i)\geq 0.75.$$
(b). The simulation result (17) is then fitted to the following parametric form:
  $$T_{f}=\frac{N^{a(T)}}{2^{b(N)}},$$
  where a(T) and b(N) are some coefficients. The following approximation, satisfying condition (17), achieves a relative error of less than 5% compared to the simulated $T_{f}$:
  (18) $$T_{f}\approx \frac{N^{\frac{1}{3}\left(\frac{T}{45}+\frac{97}{90}\right)}}{2^{\frac{\log N}{2}-4}}.$$

To better validate the results presented in Theorem 1, we visualize the probability described in the theorem. To maintain consistency with the theoretical setting, we exclude the influence of CRC, focusing solely on the ability of the threshold flipping strategy to accurately identify and flip the true FEP (the first error bit where the SC decoder fails). Additionally, we employ the GA method [18] to generate the information bit set A and ensure that the SNR required for the SC algorithm to achieve a BLER of $10^{-2}$ was minimized for different code lengths. Specifically, for code lengths N∈{1024,2048,4096,8192,16,384}, we set the corresponding design SNRs for the GA method to {2.75,2.5,2.25,2,1.85} dB, respectively.

**Figure 4:**
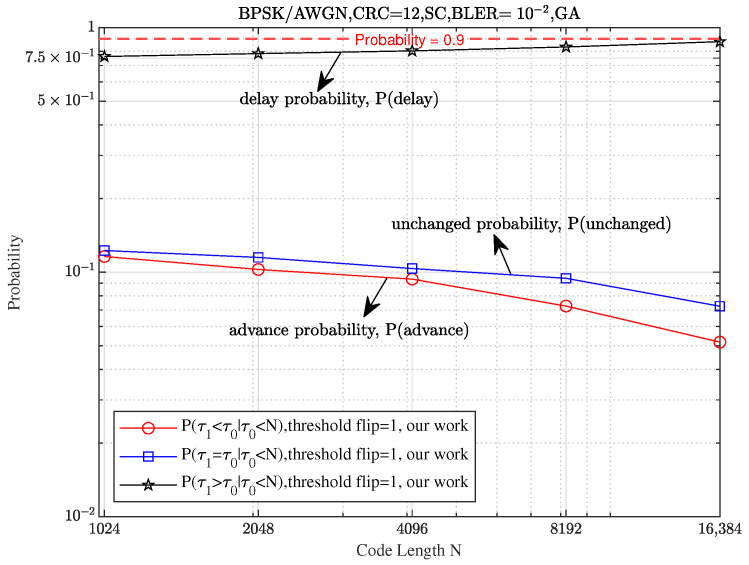
Probabilities of delay, unchanged, and advance for various code lengths N∈{1024,2048,4096,8192,16384} at a fixed code rate $R=\frac{1}{2}$ and design SNRs {2.75,2.5,2.25,2,1.85} dB, with a target BLER of $10^{-2}$, using Th-SCF algorithm (Algorithm 1) with T=1 and $T_{f}=\frac{N^{1.1/3}}{2^{\frac{\log N}{2}-4}}$.

As shown in Figure 4, the delay probability of the first error position (FEP) for the threshold SCF (Th-SCF) algorithm (represented by the black star-shaped curve) increases to 1, while the probabilities for advancement and no change (represented by the blue square-shaped curve and red circle-shaped curve, respectively) decrease to 0 as *N* increases. This indicates a significant performance improvement for long polar codes.

### 4.1. Error Correction Performance of the Proposed Th-SCF Algorithm

In this subsection, we evaluate the performance of the proposed algorithm under different parameter settings. Specifically, we compare the performance of our algorithm with existing SCF algorithms across various code lengths *N*, flipping attempts *T*, code rates *R*, and flipping thresholds $T_{f}$, highlighting the advantages of the proposed approach under these varying conditions.

**Figure 5:**
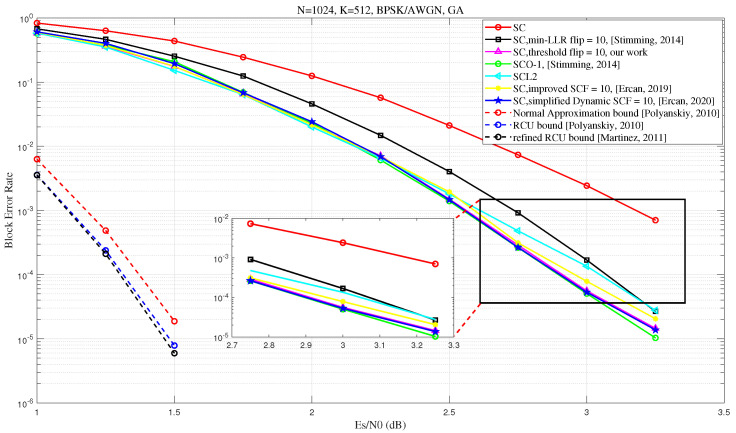
BLER performance comparison between the proposed Th-SCF algorithm and several existing SCF algorithms, including the min-LLR SCF algorithm [6], the SCO algorithm [6], the improved SCF algorithm [10], the simplified D-SCF algorithm [17], the normal approximation (NA) bound [21], the random-coding union (RCU) bound [21], and the refined RCU bound [22]. The comparison is conducted with a maximum of 10 flipping attempts ($T_{\max}=10$), a threshold value of $T\_f=\frac{1024^{1.3/3}}{2}$, and a 12-bit CRC using the generator polynomial $g\left(x\right)=x^{13}+x^{11}+x^{3}+x^{2}+1$. The information bit set A is constructed using the GA method, with a design SNR of 2.5 dB.

Firstly, we compare the BLER performance of the proposed algorithm with several existing SCF algorithms. As shown in Figure 5, as the SNR increases, our proposed algorithm achieves comparable performance with the simplified D-SCF [17] and improved SCF [10] algorithms. Furthermore, when the maximum number of flips is set to $T_{\max}=10$, our algorithm surpasses CA-SCL with list size L=2 [5], with performance approaching that of the SCO-1 decoder (Section III-C of ref. [6]) These results provide compelling evidence for the effectiveness of the proposed algorithm. While the proposed algorithm exhibits strong decoding performance, a comparison with finite-length achievability bounds—such as the Normal Approximation (NA) bound, the Random-Coding Union (RCU) bound, and the refined RCU bound [21,22]—indicates that there is still considerable room for improvement. This observation highlights the potential for further research aimed at closing the gap to the theoretical limits at finite block lengths.

**Figure 6:**
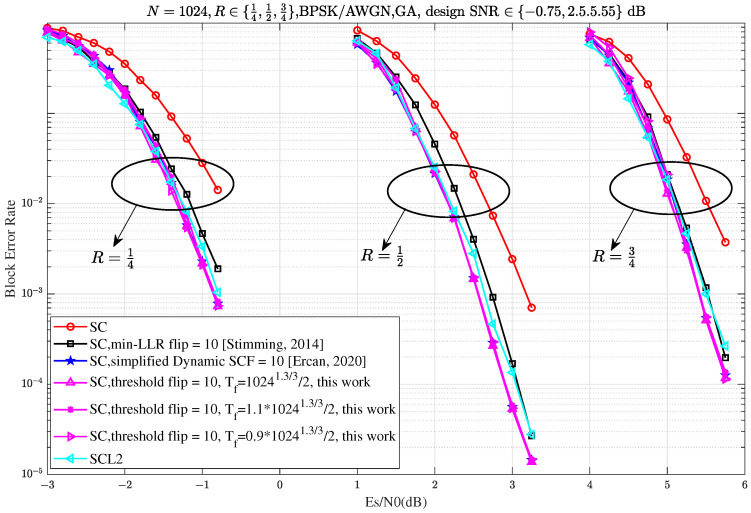
BLER performance comparison of min-LLR SCF ([6], Figure 5), simplified D-SCF [17], SC-List decoding [5] with list size L=2, and the proposed Th-SCF algorithm (this work) for polar codes of length N=1024 and code rates $R\in \{\frac{1}{4},\frac{1}{2},\frac{3}{4}\}$. All SCF-based decoders are configured with a CRC length of 12, while the SC-List decoder uses a CRC length of 8 as suggested in [10]. The maximum number of flipping attempts is set to $T_{\max}=10$, and the threshold for Th-SCF is $T_{f}=\frac{1024^{1.3/3}}{2}$.

Figure 6 demonstrates that the proposed Th-SCF algorithm (Algorithm 1) achieves comparable performance to the simplified dynamic SCF (D-SCF) decoding algorithm across different code rates. Moreover, the Th-SCF algorithm exhibits almost the same performance within a threshold range of approximately $0.9\times T_{f}$ to $1.1\times T_{f}$, where $T_{f}=\frac{1024^{1.3/3}}{2}$, which is visually supported by the three red dashed lines in the figure. Additionally, the proposed Th-SCF algorithm outperforms CA-SCL with list size L=2 after 10 flips across all tested code rates, further confirming the effectiveness and practicality of our approach.

**Figure 7:**
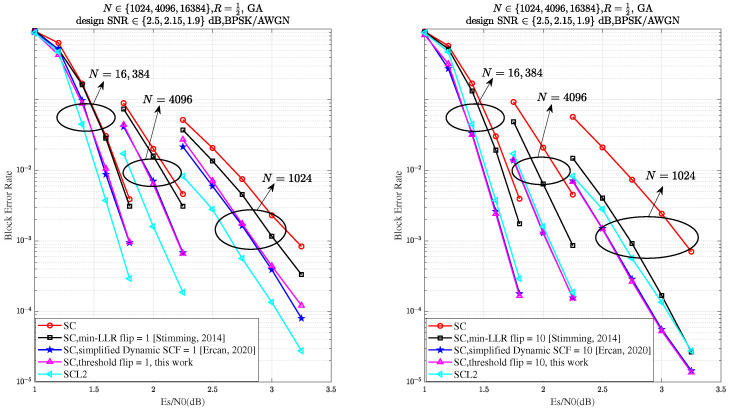
BLER performance comparison of the min-LLR SCF ([6], Figure 5), simplified D-SCF [17], SC-List [5] with L=2, and Th-SCF (this work) algorithms for code lengths N∈{1024,4096,16384}, code rate $R=\frac{1}{2}$, and corresponding design SNRs ∈{2.5,2.15,1.9} dB. The maximum number of flipping attempts *T* is set to 1 and 10, while $T_{f}$ is estimated using (18). All SCF decoders use a CRC length of 12, while the SC-List decoder uses a CRC length of 8, as recommended in [10].

As illustrated in Figure 7, the proposed Th-SCF algorithm demonstrates performance comparable to the simplified D-SCF algorithm across various flipping attempts *T* and code lengths *N*. Additionally, for all code lengths *N*, it is evident that when the number of flips reaches 10, the performance of the proposed algorithm surpasses that of CA-SCL with list size L=2, thereby validating the effectiveness of our approach.

### 4.2. Complexity Comparison with Existing SCF Algorithms and Performance Evaluation in Non-Ideal Channels

In this section, we compare the average complexity of the proposed Th-SCF algorithm with that of existing SCF algorithms. Additionally, we highlight the advantages of our proposed algorithm in non-ideal channels, such as Rayleigh fading channels, to demonstrate its practical applicability in real-world scenarios.

**Figure 8:**
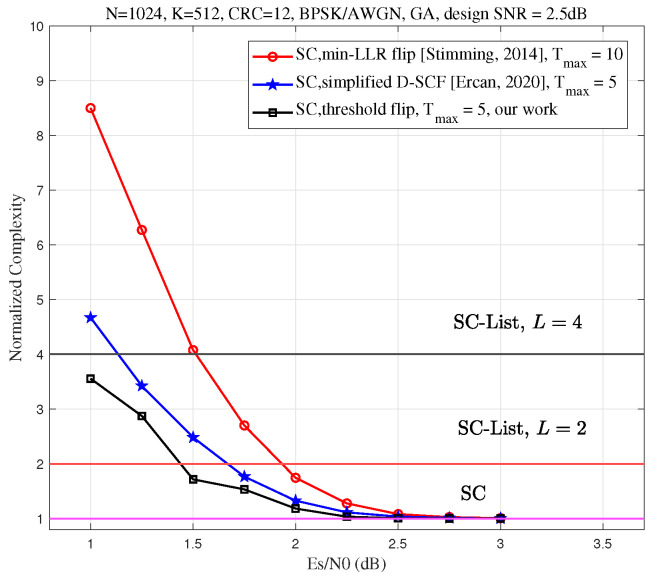
Average complexity comparison of the min-LLR SCF [6], simplified D-SCF [17], and Th-SCF (Algorithm 1) algorithms, normalized to the complexity of SC decoding, for a polar code with length N=1024, code rate $R=\frac{1}{2}$, and a 12-bit CRC. The information bit set A is generated using the GA method, with the design SNR chosen to be 2.5 dB.

In Figure 8, we compare the average complexity of the proposed Th-SCF algorithm with that of the min-LLR SCF and simplified D-SCF algorithms. For this comparison, we set the code length N=1024 and the code rate to $\frac{1}{2}$. The information bit set A is generated using the GA method, with the design SNR set to 2.5 dB. The maximum number of flips for the min-LLR SCF algorithm is fixed at 10. To ensure a fair comparison, the maximum number of flips for the Th-SCF algorithm is adjusted so that its error-correction performance matches that of the min-LLR SCF algorithm. Specifically, when the code rate $R=\frac{1}{2}$, both the Th-SCF and simplified D-SCF algorithms achieve performance comparable to that of the min-LLR SCF algorithm with 10 flips when the maximum number of flips $T_{\max}=5$.

As shown in Figure 8, in the high SNR regime, the average complexity of all algorithms converges to that of the SC decoding algorithm. However, in the low SNR regime, the proposed algorithm significantly reduces the average complexity compared to the min-LLR SCF algorithm. Additionally, when the maximum number of flips $T_{\max}$ is fixed, the complexity of the proposed algorithm is lower than that of the simplified D-SCF algorithm. This demonstrates the efficiency of the proposed algorithm and highlights its advantage in terms of reduced average complexity.

Next, we explore the practicality of the proposed algorithm in real-world applications. Since the Gaussian approximation (GA) assumption (Assumption 1) may not always hold in practical scenarios, it is essential to evaluate the performance of the proposed algorithm under non-ideal channel conditions. To this end, we consider the Rayleigh fading channel as a representative case.

**Figure 9:**
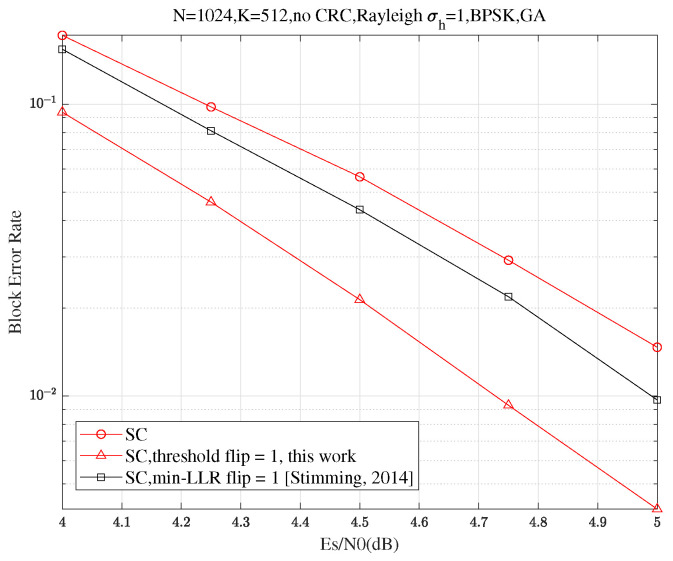
BLER performance comparison of the min-LLR SCF [6] and Th-SCF algorithm (Algorithm 1) for code length N=1024, code rate $R=\frac{1}{2}$ under Rayleigh fading, with T=1, where $h∼\mathrm{Rayleigh}(\sigma_{h})$ and $\sigma_{h}=1$. The information set A is constructed using the GA method with a design SNR of 5 dB [23].

Figure 9 compares the performance of the proposed Th-SCF decoder with the min-LLR SCF decoder as described in Figure 5 of ref. [6] over a Rayleigh fading channel where the fading coefficient h∼Rayleigh(1), evaluated within the framework of Trifonov as summarized in Figure 4 of ref. [23]. The proposed decoder achieves an approximate 0.27 dB performance gain at a BLER of $10^{-2}$, highlighting two key advantages:

1. **Practicality**: The method retains strong error correction performance even when the Gaussian approximation (GA) assumption (Assumption 1) is no longer valid, demonstrating its reliability in practical, non-ideal fading environments.
2. **Versatility**: Despite offering improved performance, it retains the efficient O(NlogN) computational complexity, making it applicable to a wider range of communication channels beyond the Gaussian case.

These results affirm the practical value and generalizability of the proposed Th-SCF decoding approach. Therefore, Assumption 1 could provide a simple (though not exclusive) threshold design, with alternative methods to be explored in future work.

### 4.3. Discussion

In this subsection, we explore potential approaches for integrating the proposed algorithm with recent techniques, such as the generalized restart mechanism (GRM) [24] and the fast decoding method [25], to further reduce its computational complexity. Both of these methods aim to enhance decoding efficiency by reducing overall complexity.

The core mechanism of the generalized restart mechanism (GRM) lies in bypassing the partial traversal of the decoding tree through strategic design, while leveraging previously stored information to estimate the bits decoded in earlier stages. This approach can be integrated with the proposed Th-SCF algorithm.

Specifically, during each iteration, we can record the index of the bit where the LLR magnitude first fails to exceed the threshold $T_{f}$ (e.g., when the bit $u_{i}$ does not meet the threshold criterion). If decoding errors remain after applying threshold-based flipping, the subsequent Th-SCF decoding iteration can preserve the decisions for bits $u_{0}$ to $u_{i-1}$, which are assumed to be correct. The decoder can then resume the SC decoding process from bit $u_{i+1}$, following the restart path defined in Definition 2 of ref. [24]. This approach effectively avoids redundant recomputation for earlier bits whose decoding outcomes remain unchanged across iterations. This is just an initial conceptual framework and more detailed design strategies will be presented in our future work.

The integration of the Th-SCF algorithm with fast decoding techniques relies on establishing appropriate threshold selection rules tailored to various node types, such as $R_{0}$, $R_{1}$, REP, and SPC nodes.

For $R_{0}$ nodes, which consist entirely of frozen bits [25], bit flipping is unnecessary. In $R_{1}$ nodes, composed exclusively of information bits [25], the bit corresponding to the smallest LLR magnitude at the top node is considered for flipping, provided its magnitude does not exceed a predefined threshold $T_{f}$.

For REP nodes, where the LLR of the single non-frozen bit is obtained by summing the LLRs of its constituent top-node bits [25], the entire REP node is subjected to flipping evaluation if this aggregated LLR magnitude falls below $T_{f}$.

The threshold determination for SPC nodes is more complex, due to their hybrid structure involving a frozen bit alongside several $R_{1}$ bits [25]. A viable approach is to incorporate the parity-based flipping criteria proposed in Equations (17a)–(18b) of ref. [26], which consider the three smallest LLR magnitudes for decision-making.

Note that the current design is only a preliminary discussion, and more detailed content will be provided in future work. We will further explore efficient strategies for implementing the fast threshold-based SCF algorithm and investigate additional node types, such as REP-SPC nodes [26], as well as refine the threshold selection techniques for optimal performance.

It is also important to emphasize that the theoretical analysis presented in this work relies on the validity of the Gaussian approximation (GA) assumption (Assumption 1). Moreover, the performance of the proposed algorithm may be affected by the choice of code construction method. As highlighted in Theorem 1, the effectiveness of the Th-SCF algorithm is influenced by several critical factors, including code length, SNR, and the specific method used to construct the information set. In particular, adopting alternative construction techniques—such as Reed–Muller (RM)-based schemes [27]—may lead to different performance outcomes. While a detailed investigation of this aspect is not provided in the current work, we acknowledge that understanding the impact of various construction methods is essential for the practical deployment of the proposed algorithm. We plan to explore this topic more thoroughly in our future work. These dependencies not only delineate the boundaries of the current study but also point to promising avenues for further research.

## 5. Conclusions

In this paper, we introduce the threshold successive cancellation flip (Th-SCF) decoding algorithm for polar codes. The Th-SCF algorithm delivers performance comparable to the dynamic SCF (D-SCF) decoder while significantly reducing computational complexity by eliminating the need for expensive sorting operations. Through rigorous theoretical analysis and extensive simulations, we show that Th-SCF can asymptotically delay the first error position (FEP) with probability 1, ensuring high decoding performance. Additionally, the proposed method demonstrates reliable performance under non-ideal conditions, such as Rayleigh fading channels, highlighting its practical applicability in real-world communication systems. Looking ahead, future work will focus on developing adaptive threshold selection strategies for non-Gaussian channels, particularly for emerging 5G and 6G networks. Furthermore, we plan to explore the integration of Th-SCF with other advanced techniques, such as the generalized restart mechanism (GRM), fast decoding methods, and iterative decoding frameworks, with the goal of further reducing computational complexity and enhancing system flexibility.

## Data Availability

The data that support the findings of this research are available from the corresponding author upon reasonable request.

## Abbreviations

The following abbreviations are used in this manuscript:

SC	successive cancellation
SCL	successive cancellation list
SCF	successive cancellation flip
Th-SCF	threshold successive cancellation flip
LLR	log-likelihood ratio
PDF	probability density function
CDF	cumulative distribution function
FEP	first error position
BPSK	binary phase shift keying
BI-AWGN	binary input additive white Gaussian distribution
CRC	cyclic redundanct check
D-SCF	Dynamic successive cancellation flip
GRM	generalized restart mechanism
RCU	Random-Coding Union
NA	Normal Approximation
